# Isolation and evaluation of proteolytic actinomycete isolates as novel inducers of pearl millet downy mildew disease protection

**DOI:** 10.1038/srep30789

**Published:** 2016-08-08

**Authors:** Sudisha Jogaiah, Mahantesh Kurjogi, Sharathchandra Ramasandra Govind, Shekar Shetty Huntrike, Vedamurthy Ankala Basappa, Lam-Son Phan Tran

**Affiliations:** 1 Plant Healthcare and Diagnostic Center, Department of Studies in Biotechnology and Microbiology, Karnatak University, Dharwad 580 003, Karnataka, India; 2 Department of Studies and Research in Environmental Sciences, Tumkur University, Tumkur 572103, Karnataka, India; 3 Downy Mildew Research Laboratory, Department of Studies in Biotechnology, University of Mysore, Manasagangotri, Mysore 570006, India; 4 Plant Abiotic Stress Research Group & Faculty of Applied Sciences, Ton Duc Thang University, Ho Chi Minh City, Vietnam; 5 Signaling Pathway Research Unit, RIKEN Center for Sustainable Resource Science, 1-7-22, Suehiro-cho, Tsurumi, Yokohama 230-0045, Japan

## Abstract

Native endophytic actinomycetes isolated from pearl millet roots were examined for their efficacy to protect pearl millet against downy mildew. Nineteen of 39 isolates were found to be proteolytic, of which 7 strains could directly suppress the sporangium formation of *Sclerospora graminicola*, the pearl millet downy mildew pathogen. Thus, mycelial suspensions containing either spores or cell-free extract of these 7 isolates were used for seed-coating and -soaking treatments to test for their induction of downy mildew resistance. Results indicated that seed-coating overall provided better protection to downy mildew than seed-soaking. In both treatments, the tested isolates demonstrated differential abilities in downy mildew disease protection, with *Streptomyces griseus* SJ_UOM-07-09 and *Streptosporangium roseum* SJ_UOM-18-09 showing the highest protection rates. Additionally, the levels of disease protection conferred by the actinomycetes were just slightly lower than that of the systemic fungicide Apron, suggesting their effectiveness. Further studies revealed that the more rapid root colonization by SJ_UOM-18-09 resulted in faster and higher induced resistance in comparison with SJ_UOM-07-09 under greenhouse conditions, indicating that SJ_UOM-18-09 was superior than SJ_UOM-07-09 in inducing resistance. Results from this study provide comprehensive information on biocontrol functions of SJ_UOM- 18-09 with great potential to control downy mildew disease in pearl millet.

Actinomycetes are not only known for their ability to produce antibiotics but also as soil microbes that influence plant growth and protects plants against pathogenic fungi^1^,^2^. More recently, endophytic actinomycetes have been reported to qualitatively and quantitatively impact the host through beneficial responses to environmental stimuli^3^,^4^. Endophytic actinomycetes, when in association with their host plants, can have many effects on them, such as enhancement of resistance against various environmental stresses, insects and diseases, as well as improvement of plant growth and productivity and exhibiting herbicide activities^5^. The colonization and propagation of endophytes and their secondary metabolites inside the plants may be critical for these effects. These facts indicate that endophytes can be potential biological control agents and play an important role in plant disease control^6^,^7^.

Actinomycetes are saprophytic in nature and decompose naturally occurring organic substrates, including lignocelluloses^8^. They are able to secrete a number of proteolytic enzymes and metabolites that enable them to degrade complex substrates, including pathogens, and are therefore novel targets in the next generation search for efficient biomass deconstruction agents^9^. Actinomycetes, when present as endophytes, have an impact on plant growth and development due to their ability to improve growth of plants by enhancing nutrient assimilation and producing volatile secondary metabolites^7^,^10^. Examples include endophytic *Streptomyces* spp. that can reduce the negative effects of fungal diseases of banana (*Musa paradisiaca*), rice (*Oryza* sativa) and wheat (*Triticum aestivum*)^2^,^11^.

Downy mildew disease caused by *Sclerospora graminicola* is responsible for worldwide yield losses on pearl millet (*Pennisetum glaucum*)^12^. The disease affects huge population of poor people of semi-arid tropic regions of Africa and Asia who rely on pearl millet for their basic sustenance. It is estimated that 50% of the world millets is occupied by pearl millet crop, and India alone accounts for more than 50% of the global output^13^. Successful control of the downy mildew disease is dependent on expensive chemicals and host resistance^14^. Due to the distinct physiological and phylogenetic niches that these organisms occupy, most of the control measures have failed^15^.

Various types of induced resistance have been reported to control crop diseases^14^,^16^,^17^. Advantages of such methods include broad spectrum resistance, thereby enhancing the crop yield in an eco-friendly and feasible manner. A number of potential inducers with the ability to suppress downy mildew disease of pearl millet have been documented in literature^13^,^18^. Nevertheless, the resistance obtained under epiphytotic conditions is dependent on various factors. However, under field conditions resistance levels are influenced by a number of factors, including host responses and prevailing environmental conditions. Therefore, it is important that the bio-agents from native soils with an array of beneficial traits^17^,^19^,^20^,^21^ on crop improvement should be exploited.

In this context, the present work was undertaken to isolate and test the efficacy of proteolytic actinomycete isolates in inducing protection against downy mildew of pearl millet. We have isolated 39 actinomycete isolates from pearl millet root samples with the aim to identify isolate(s) that could provide significantly enhanced resistance of pearl millet to downy mildew. The isolated actinomycetes were then characterized for their beneficial characteristics that enable them to confer protection against downy mildew caused by *S. graminicola*.

## Results

### Classification of identified endophytic actinomycete strains isolated from pearl millet roots using *16S* rRNA sequencing and morphological assays

A total of 47 isolates were obtained from roots of the pearl millet cv. 1P18192 that has been known to be resistant to downy mildew disease^18^. As a next step in our pipeline, the *16S* rRNA sequencing method was used to further classify these 47 endophytic isolates. The *16S* rRNA genes from all 47 actinomycete strains yielded PCR fragments with size between 1.10–1.45 kb. These fragments were sequenced, and the sequence data were subjected to a homology analysis using the BLASTN. The results obtained by *16S* rRNA sequencing revealed that among the 47 endophytic isolates 39 strains were found to belong to actinomycete group (Table 1). Specifically, the nucleotide sequences of 29 *16S* rRNAs showed overall similarity scores between 87 to 99% to those of the genus *Streptomyces* (group I) which was further divided into 4 subgroups (from I to IV) with 9, 8, 6 and 6 isolates, respectively, while the *16S* rRNA sequences of the remaining 10 isolates showed homology (90–98% similarity) to those of *Streptosporangium* spp. (group II) that could also be classified into 4 subgroups (from I to IV) of 4, 3, 2 and 1 isolates. Furthermore, among the 29 *Streptomyces* isolates of group I, *16S* rRNA sequences of the members of subgroups I, II and III exhibited high degree of similarity to those of *S. griseus* (91–99% sequence similarity), *S. coelicolor* (87–98% sequence similarity) and *S. verticillus* (93–99% sequence similarity), respectively, while those of subgroup IV did not display similarity to any specific species. Thus, the 6 members of subgroup IV were identified only up to their *Streptomyces* genus level. With regard to group II of *Streptosporangium* spp., members of subgroups I, II, III and IV isolates possess *16S* rRNA sequences similar to *S. amethystogenes* (90–98% sequence similarity), *S. vulgare* (91–97% sequence similarity), *S. roseum* (90–93% sequence similarity) and *S. shengliense* (93% sequence similarity), respectively. All the isolates were purified and internal catalogue number was assigned to each strain (Table 1).

Next, all the 39 actinomycete isolates were subjected to various morphological assays to obtain an overview about their morphological characteristics. Similar to the results of *16S* rRNA sequencing, the 39 isolates could also be differentiated into 2 major morphological groups I and II, each of which consisted of 4 subgroups, on the basis of their characteristics displayed on the ‘S’ medium, including color of aerial mycelia, pigment production and morphology of spore chains (Table 1).

### Growth on nitrogen (N)-free media, siderophore production and proteolytic activity of the endophytic actinomycetes

Growth abilities of 39 isolates were assayed on agar plates containing N free medium (NFB) or N-low medium (NLB). Thirty-two of 39 isolates demonstrated growth on the above media. These 32 isolates were further tested for their ability to produce siderophores, of which 23 strains were found to produce siderophores as evidenced by the pinkish brown color observed on plates containing chrome azurol S (CAS) medium. Two strains, *Streptomyces griseus* SJ_UOM-07-09 and *Streptosporangium roseum* SJ_UOM-18-09, recorded the highest amount of siderophores (Table 2).

**Figure 1:**
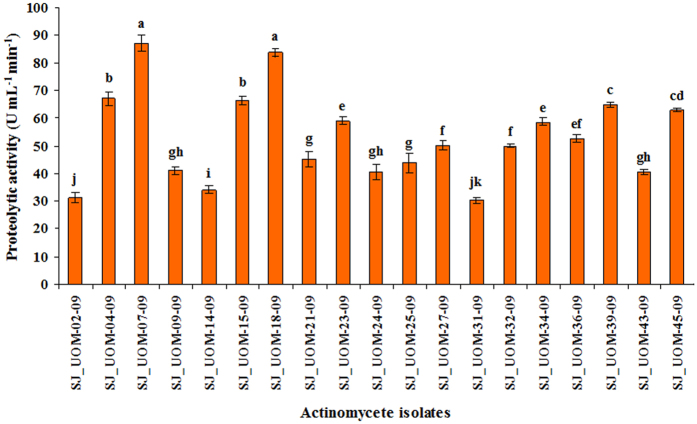
Proteolytic activity of cell-free extracts (CFEs) of actinomycetes isolated from pearl millet roots. Data of 19 isolates are shown as other strains were found to be negative for proteolytic activity. Values are means ± standard errors (SEs) of four independent replications (n = 4). Bars represent SEs. Different letters within the column indicate statistically significant differences according to Scheffe’s post hoc test (*P* < 0.05).

In addition, among the 23 siderophore-producing isolates, 19 showed positive proteolytic activity (Fig. 1). SJ_UOM-07-09 and SJ_UOM-18-09 exhibited similar levels of proteolytic activity, which were the highest when compared with those produced by the remaining strains. Cell-free extracts (CFEs) of all other actinomycete isolates also exhibited proteolytic activity, although at lower levels, ranged from 30.3 units (*Streptosporangium vulgare* SJ_UOM-31-09) to 67 units (*Streptosporangium amethystogenes* SJ_UOM-04-09). Only the isolates, which exhibited proteolytic activity, were used in further studies.

### *In vitro* effects of CFEs of actinomycete isolates on *S. graminicola* sporangial formation and zoospore release from sporangia

**Figure 2:**
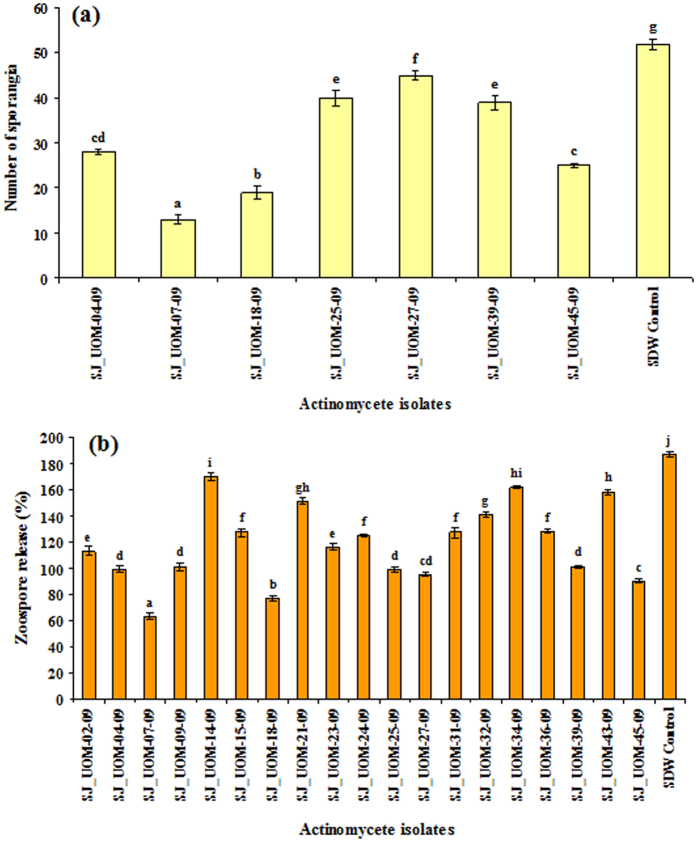
Anti-mildew activity of tested actinomycete isolates. **(a)** Number of sporangia of *Sclerospora graminicola* per cm^2^ of the leaf. **(b)** Percent zoospore release from sporangia after treatment with cell-free extracts (CFEs) of actinomycete isolates. Values are means of four independent replications. Bars represent standard errors. Different letters indicate statistically significant differences according to Scheffe’s post hoc test (*P* < 0.05). SDW, sterile distilled water.

The formation of sporangia and percentage of zoospores released from sporangia were examined after treatment of infected leaves with CFEs of all 19 actinomycete isolates that were proteolytic. Among the 19 tested isolates, only 7 were capable of inhibiting the sporangial production, suggesting that these 7 isolates possess a direct anti-mildew activity (Fig. 2a). The decrease in the sporangial number differed after treatment with different strains. On the other hand, all the 19 tested proteolytic actinomycete isolates recorded fair to good inhibition of zoospore release in comparison with the sterile distilled water (SDW) control (Fig. 2b). SJ_UOM-07-09 and 18-09 demonstrated the highest anti-mildew effects when compared with other tested strains. Seven strains, namely *Streptosporangium amethystogenes* SJ_UOM-04-09 and 39-09, *Streptomyces griseus* SJ_UOM-07-09, *Streptosporangium roseum* SJ_UOM-18-09, *Streptomyces verticillus* SJ_UOM-25-09 and 45-09 and *Streptomyces* sp. SJ_UOM-27-09, which could inhibit both host-dependent sporangial production (Fig. 2a) and host-independent zoospore release (Fig. 2b), were selected for our next study to find out whether they could provide effective protection to pearl millet plants against *S. graminicola* infection by induction of systemic protection.

### Downy mildew disease control by the proteolytic actinomycetes under greenhouse conditions

**Figure 3:**
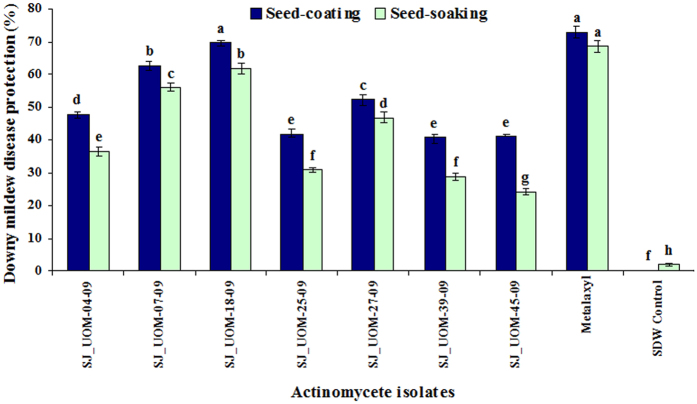
Efficacy of seed treatment with actinomycetes on downy mildew disease. The evaluation was made 30 days after sowing. Values are means ± standard errors (SEs) of four independent replications (n = 4) conducted under greenhouse conditions. Bars represent SEs. Different letters within the column indicate statistically significant differences according to Scheffe’s post hoc test (*P* < 0.05). SDW, sterile distilled water.

The effects of the 7 proteolytic actinomycetes in mitigating downy mildew were tested using both seed-coating and seed-soaking methods. The highest downy mildew disease protection rates, after artificial inoculation of the pathogen to the seedlings, were noted for SJ_UOM-07-09 and 18-09 strains that offered 62.5 and 69.4% protection rate, respectively, when treated as seed-coating (10^8^–10^10^ spores mL^−1^). On the other hand, seeds coated with SJ_UOM-39-09 strain showed the lowest disease protection of 40.5% (Fig. 3). For the same strains SJ_UOM-07-09 and 18-09, 56 and 61.7% downy mildew disease protection rates, respectively, were recorded when they were used for seed-soaking, which were also the highest protection rates as compared with those provided by the remaining 5 strains. The lowest disease protection of 24.1% was noted with SJ_UOM-45-09 strain treated in the form of seed-soaking. Metalaxyl, which was used as the standard chemical control, provided 72.9 and 68.6% downy mildew disease protection in seed-coating and -soaking treatments, respectively. However, water controls used in the two treatments were unable to protect pearl millet against the downy mildew disease after pathogen challenge. Our results collectively indicated that seed-coating with the mycelial suspension (10^8^–10^10^ spores mL^−1^) of the proteolytic actinomycetes offered more protection against downy mildew disease to pearl millet plants than seed-soaking treatment with their CFEs. Furthermore, the data demonstrated that among the 7 tested isolates, SJ_UOM-18-09, a *Streptosporangium roseum*, was the best strain for plant protection, followed by SJ_UOM-07-09, a *Streptomyces griseus*, in both two treatment methods. Thus, these two strains were selected for further studies.

### *In planta* colonization of *Streptomyces griseus* SJ_UOM-07-09 and *Streptosporangium roseum* SJ_UOM-18-09

**Figure 4:**
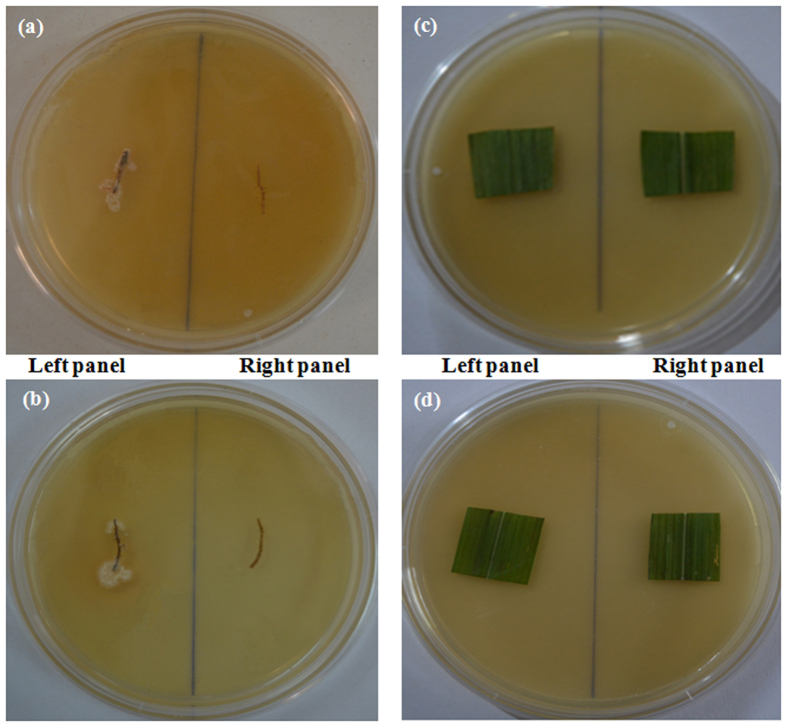
*In planta* root colonization of *Streptomyces griseus* SJ_UOM-07-09 and *Streptosporangium roseum* SJ_UOM-18-09. Surface-sterilized roots of one-month-old pearl millet raised from seeds coated with (**a**) SJ_UOM-07-09 and (**b**) SJ_UOM-18-09 showed the profuse growth of the strains on ‘S’ medium after 12 and 9 days of incubation, respectively (left panel). Surface-sterilized leaves of one-month-old pearl millet raised from seeds coated with (**c**) SJ_UOM-07-09 and (**d**) SJ_UOM-18-09 displayed no growth of the strains on ‘S’ medium after 12 days of incubation (left panel). (**a–d**; right panel) Roots and leaves of plants raised from seeds treated with sterile distilled water exhibited no growth of the bacteria.

*Streptomyces griseus* SJ_UOM-07-09 and *Streptosporangium roseum* SJ_UOM-18-09 that were shown to the most significantly enhance the pearl millet downy mildew disease protection were used in this study. Surface-sterilized roots of plants raised from seeds coated with SJ_UOM-07-09 or SJ_UOM-18-09 showed luxuriant growth of the actinomycetes after 9 and 12 days of incubation (Fig. 4a,b; left panel), respectively, suggesting that SJ_UOM-18-09 may possess more efficient root colonization ability than 07-09. No growth of SJ_UOM-07-09 or SJ_UOM-18-09 was noted either in the pearl millet leaves harvested from plants raised from the seeds primed with these two actinomycetes (Fig. 4c,d; left panel). Additionally, no growth was observed in the control set of roots or leaves that were sampled from plants raised from seeds treated with SDW (Fig. 4a–d; right panel). Taken together, our results indicated that both SJ_UOM-07-09 and 18-09 could internally colonize the indigenous host roots, demonstrating their ability of endophytic association which might lead to enhancement of induced resistance to protect plants from diseases.

### Proteolytic *Streptomyces griseus* SJ_UOM-07-09 and *Streptosporangium roseum* SJ_UOM-18-09 require a time gap for the build-up of downy mildew disease protection

**Figure 5:**
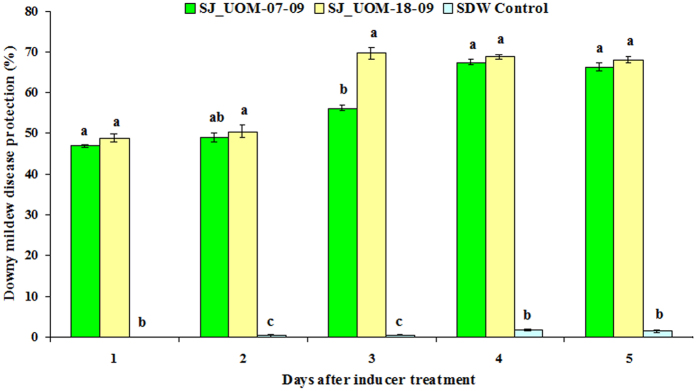
Seed-coating with SJ_UOM-07-09 and SJ_UOM-18-09 strains and their spatio-temporal effects during induction of resistance. The evaluation was made 30 days after sowing. Values are means ± standard errors (SEs) of four independent replications (n = 4) conducted under greenhouse conditions. Bars represent SEs. Different letters within the column indicate statistically significant differences between treatments and sterile distilled water (SDW) control, as measured by Scheffe’s post hoc test (*P* < 0.05).

**Figure 6:**
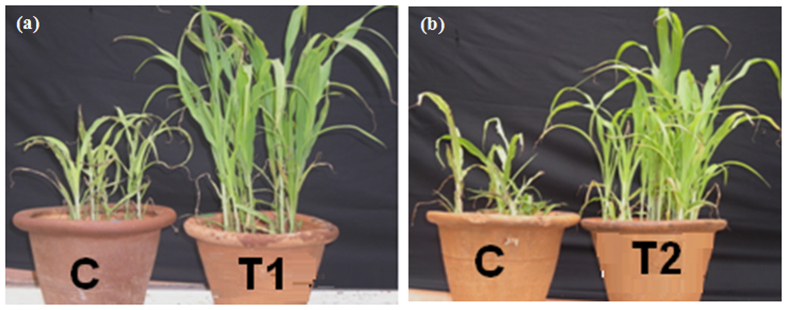
Representative image showing induced resistance by seed-coating with SJ_UOM-07-09 and SJ_UOM-18-09. Images of (**a**) *Streptomyces griseus* SJ_UOM-07-09 (T1) and (**b**) *Streptosporangium roseum* SJ_UOM-18-09 (T2) stimulating induced protection to pearl millet downy mildew disease were captured at 30-days after sowing from experiments with 4 and 3 days of time gap period, respectively. “C” represents the sterile distilled water-treated control plants expressing typical symptoms of downy mildew disease, such as stunted growth, chlorosis of leaf, cottony growth on lower leaves and necrosis on leaf tips.

Efforts were then made to examine whether the *in planta* colonization of SJ_UOM-07-09 and 18-09 strains could provide protection against *S. graminicola* through induced resistance by maintaining time gap between inducer treatment and pathogen challenge. We found that the protection of 30-day-old pearl millet plants against downy mildew disease conferred by SJ_UOM-07-09 and 18-09 after seed-coating treatment increased progressively. In SJ_UOM-18-09-treated plants inoculated with the pathogen, downy mildew disease protection was 48.8% after day 1 (5-day-old plants after sowing), and gradually increased to 50.5, 69.7, 68.9 and 68.2% with 2 (6-day-old plants after sowing), 3 (7-day-old plants after sowing), 4 (8-day-old plants after sowing) and 5 days (9-day-old plants after sowing) of time gap period, respectively (Fig. 5). The same trend was also observed for SJ_UOM-07-09 with different protection margins, which recorded the highest protection rate of 67.5% at day 4 (8-day-old plants after sowing) after challenge-inoculation (Fig. 5). These results indicated that SJ_UOM-18-09 expressed a significantly earlier and higher induction of resistance than SJ_UOM-07-09, requiring 3 days of time gap period compared with 4 days for SJ_UOM-07-09, after challenge-inoculating the pearl millet seedlings with *S. graminicola*, for developing maximum protection (Fig. 5). This finding thus demonstrated that the induced systemic resistance observed through the endophytic colonization of the pearl millet roots with SJ_UOM-18-09 by seed coating is superior in comparison with SJ_UOM-07-09 in protecting pearl millet plants against *S. graminicola* infection (Fig. 6a,b).

## Discussion

In the present study, we have obtained 47 endophytic isolates from roots of the downy mildew-resistant pearl millet cv. 1P18192^18^ using selective isolation steps. The taxonomic identity of the isolated strains was then identified by the means of *16S* rRNA sequencing and their culture morphology. The *16S* rRNA sequencing results of 47 isolates enabled us to identify 39 actinomycete isolates. Additionally, both the results of *16S* rRNA sequencing and morphological assays further classified these 39 isolates into 2 major groups; each had 4 subgroups, up to the genus and/or species levels with high degree of confidence (Table 1).

Out of these 39 isolates, 32 showed tendency to grow well without N source, indicating their N_2_-fixing ability (Table 2). Further evaluation indicated that 23 of these 32 N_2_-fixing isolates were also positive for siderophore production on the CAS medium as indicated by appearance of the pinkish brown color (Table 2). Production of siderophores by the actinomycetes indicates their ability to bind iron by chelation, which might be an important factor for actinomycetes to control soil-borne plant diseases. It has been shown by Gopalakrishnan^22^ that the rhizosphere actinomycetes produce siderophores that selectively bind iron presented in the soil, thereby competitively reducing the iron available for pathogens, which leads to suppression of pathogen growth. Therefore, our study of isolating siderophore-producing endophytic actinomycetes might have potential for field application.

To be used as a potential biocontrol agent, a particular strain should demonstrate multiple mechanisms of controlling pathogen growth and spread. In addition to production of siderophores, proteolytic activity is also one of the major attributes of endophytic actinomycetes. Since actinomycetes like *Streptromyces* have been shown to be very efficient in proteolysis^23^, it is expected that the secreted proteolytic enzymes might hydrolyze membranes of zoospores. Such an activity has been reported against *Phytophthora sojae*^24^, an oomycete pathogen. Our results show that 19 of 23 strains (representing 82.6%), which produced siderophores, also synthesized proteolytic enzymes (Fig. 1). Proteolysis of the zoospores might directly inhibit the pathogen by preventing sporangial production. We observed that out of 19 proteolytic isolates, seven strains were able to inhibit host-dependent sporangial production (Fig. 2a). Interestingly, all 19 proteolytic strains were able to inhibit host-independent zoospore release (Fig. 2b). This inhibitory action of these isolates on *S. graminicola* revealed that the tested strains can be used as novel agents that have potential in not only triggering host resistance but also suppressing the growth of pathogen by antagonistic activity. Additionally, two of 7 isolates, namely *Streptromyces griseus* SJ_UOM-07-09 and *Streptrosporangium roseum* SJ_UOM-18-09, demonstrated both stronger host-dependent and -independent anti-mildew activities than the remaining 5 strains (Fig. 2a,b), presumably because of their ability to act via diverse mechanisms, including their potential to produce anti-mildew compounds with higher bioactivities, as evidenced by their higher siderophore production (Table 2) and proteolytic activity (Fig. 1). Our results are in line with a recent study, in which 70% inhibition of mycelia of the oomycete pathogen *P. infestans* was successfully achieved by the siderophore-producing actinomycete strain ATMY-1 isolated from Indian soil^25^. The same strain was also found to be effective in suppressing the tomato (*Solanum lycopersicum*) rot fungus *Sclerotium rolfsii* by 60%^25^. Reports on antagonist nature of actinomycetes with different modes of action in plants, such as pistachio (*Pistachio orchards*), chickpea (*Cicer nigrum*), soybean (*Glycine max*), cucumber (*Cucumis sativa*), red pepper (*Capsicum annuum*) and sugar beet (*Beta vulgaris*), against different phytopathogens, such as *P. drechsleri, F. oxysporum* f. sp. *ciceri*, *Phytophthora sojae*, *Pythium aphanidermatum*, *P. capsici* and *S. rolfsii* have been widely published^22^,^24^,^26^,^27^,^28^,^29^, supporting that actinomycetes possessing bioactive compounds like siderophore and proteolytic enzymes are useful biocontrol agents with multiple benefits.

Among the various methods tested, seed treatment is the only effective and economical method for downy mildew control^13^, because pearl millet is cultivated by most economically backward farmers across the globe. Because of various market factors and low profit return, these farmers cannot afford expensive and repeated control measures^13^,^14^,^20^. Thus, there is not only the need for finding newer and more effective inducers of resistance against downy mildew disease, but also for optimizing the method(s) by which new inducers of resistance are made viable, durable, robust and economical. In order to meet such a criteria, in the present study susceptible pearl millet seeds (HB3) were primed with 7 antagonistic actinomycete isolates in the form of seed-coating or seed-soaking treatment (Fig. 3). The results from our study clearly indicate that with all the tested strains, seed-coating (spores) offered better protection than the seed-soaking (CFE) treatment. Furthermore, we noticed that SJ_UOM-18-09 and SJ_UOM-39-09 provided the highest and lowest protection, respectively, by seed-coating treatment (Fig. 3). According to our results, the differential disease protection provided by seed-coating and -soaking treatment can be explained by the fact that the application of spores of beneficial microbes, including those of actinomycetes, through seed-coating forms a protective layer all around the seed coat, allowing an early colonization in the roots of germinated seeds which improves germination^30^,^31^. Such an association could be hypothesized to activate natural plant resistance mechanisms involving translocation of plant signals from roots, thereby increasing the capacity of plant defenses against multiple pathogens^32^. Another possibility is that microbial spores are more robust and resistant to the extreme conditions, and thus can actively compete with the phytopathogens^7^,^33^. Moreover, the shelf-life of microbial spores-based biological products can last for 1–3 years, which can thus effectively be used as a stable biological agent^33^.

It has been shown that the potential of the biocontrol microbes to reduce soil-borne pathogens is related to its efficiency in colonizing the host roots^34^,^35^. Such internal colonization by the inducer is therefore responsible for development of resistance and crop improvement^34^,^35^,^36^,^37^. The current findings also revealed a positive correlation between the root colonization and the biocontrol ability. We observed that SJ_UOM-18-09 exhibited a more rapid root colonization (3 days earlier) than SJ_UOM-07-09 (Fig. 4a,b; left panel). Consistent with this noted faster colonization, an earlier and higher induction of resistance against downy mildew disease was witnessed in plants raised from seeds coated with SJ_UOM-18-09 in comparison with SJ_UOM-07-09 (Fig. 5). Similar tendency of correlation showing the efficacy of root colonization properties of other actinomycetes in contributing to induced systemic resistance against the damping-off disease in tomato^36^ and the damping-off and crown root rot^38^ in cucumber, has been previously documented. Furthermore, this correlation can be defined as the ability of microbes that are known to produce antibiotics and compete for essential nutrients and niches with pathogens, thereby suppressing the growth and development of the pathogens, leading to induce protection^35^,^39^. Other prominent examples include the work of Kurth *et al.*^5^ which showed induced systemic resistance to powdery mildew infection in pedunculate oak (*Quercus robur*) plants after treatment with *Streptomyces* sp. AcH 505 by triggering plant defense signals. Mei-Xia *et al.*^40^ reported that tomato seedlings treated with the *S. microflavus* DK56 induced effective protection against pepper blight disease caused by *P. capsici*. Most recently, Dalal and Kulkarni^41^ demonstrated that seed treatment with various actinomycetes significantly inhibited the growth of *R. solani* and protected soybean plants against *R. solani* as a result of induced protection. Hence, it is reasonable to presume that the siderophore-producing and N_2_-fixing endophytic actinomycete inoculants like those used in our study (Table 2) have the ability to escape competition with those of plant growth-affecting rhizosphere as well as saprophytic microbes by colonizing the internal tissues of the host plant^42^, which might be responsive for the enhanced plant protection (Figs 3, 5 and 6). In addition, the highest proteolytic activties observed in the SJ_UOM-07-09 and SJ_UOM-18-09 strains (Fig. 1), might also contribute to their most effective inhibition of *S. graminicola* by degrading its cell wall/membranes, which indirectly reduced disease incidence (Figs 2 and 3) as also supported by earlier studies^3^,^7^,^36^.

In conclusion, we have noticed that with regard to the actinomycete strains isolated in the present study, seed-coating treatment is more effective than seed-soaking approach in providing resistance against the pearl millet downy mildew pathogen *S. graminicola. Streptosporangium roseum* SJ_UOM-18-09 was identified as the best strain followed by *Streptomyces griseus* SJ_UOM-07-09 in providing disease protection to pearl millet against downy mildew disease. Thus, both strains play a role as novel biological agents by possessing anti-mildew properties with proteolytic activity and siderophore production. Additionally, the slightly higher resistance obtained with SJ_UOM-18-09 in comparison with SJ_UOM-07-09 can be a synergistic effect of both antagonism and induced resistance through internal colonization ability of SJ_UOM-18-09, which makes this strain favorable for exploitation as a commercial formulation, providing farmers with an affordable and eco-friendly alternative measure for stable pearl millet downy mildew disease control.

## Methods

### Plant materials

Seeds of susceptible pearl millet cultivar HB3 were received from All India Coordinated Pearl Millet Improvement Project (AICPMIP), while those of resistant pearl millet cultivar 1P18192^18^ were obtained from ICRISAT, Patancheru, India.

### Pathogen and inoculum preparations

Zoospores of *S. graminicola* were harvested from susceptible host under greenhouse conditions (22 ± 2 °C and 90% relative humidity). Briefly, leaves showing infection were washed in order to remove remaining sporangia. Blot-dried leaves were placed in humid Petri plates and sporangia were collected into SDW. The zoospore concentration was adjusted to 4 × 10^4^ zoospores mL^−1^ using a haemocytometer^13^.

### Isolation of the endophytic actinomycetes

Root samples collected from resistant pearl millet 1P18192 cultivar grown under field conditions were surface-sterilized as previously described in Cao *et al.*^6^. Ten root fragments were placed on plates containing ‘S’ medium composed of malt extract 10 g L^−1^, yeast extract 4 g L^−1^, dextrose 4 g L^−1^, CaCO_3_ 0.02 g L^−1^ and agar 15 g L^−1^, and supplemented with 25 ppm K_2_Cr_2_O_4_ and 15 ppm nalidixic acid to suppress opportunistic bacteria and fungi^43^. These plates were incubated at 27 °C for 17 days. Edges of developing colonies were transferred onto fresh ‘S’ medium and incubated for 6–8 days. The procedure was continued until eventual pure cultures were obtained.

### Classification of the endophytic actinomycetes

The identity of the endophytic isolates was ascertained by sequencing the *16S* rRNA genes of all 47 isolates, which were obtained by polymerase chain reaction (PCR) using the conserved primers (F: 5′-ACAAGCCCTGGAAACGGGT-3′ and R: 5′-ACGTGTGCAGCCCAAGACA-3′)^43^. The thermal profile of the PCR was 94 °C for 5 min, 35 cycles of 94 °C for 60 s, annealing at 59 °C for 30 s, 72 °C for 90 s and final extension at 72 °C for 7 min using the C1000 thermal cycler (Bio-Rad, USA). The PCR products were directly sequenced using the BigDye Terminator v3.1 Cycle Sequencing Kit (Applied Biosystems, USA) and the ABI 3130 automated DNA sequencer (Applied Biosystems, USA). Sequence assembly was performed using Finch TV version 1.4.0 (www.geospiza.com). The sequence data of 39 identified actinomycete isolates were deposited in the GenBank (http://www.ncbi.nlm.nih.gov/genbank/) developed by the National Center for Biotechnology Information (NCBI) under the accession numbers from KX139471 to KX139509. Homology levels of the *16S* rRNA sequences of the isolates were analyzed using BLASTN program from the GenBank database. Furthermore, the identified *16S* rRNA strains were also subjected to culture morphological assays that included color of aerial mycelia, pigment production and morphology of spore chains as described by Shirling and Gottlieb^44^ and Goodfellow and Cross^45^.

### Growth on N-free media

The growth of the actinomycetes was compared on plates containing (i) NFB or (ii) NLB according to Franco-Correa *et al.*^46^. The compositions of the NFB and NLB media are as follows: (i) NFB (D-malic acid 0.5%, NaCl solution (10%) 1 mL L^−1^, KH_2_PO_4_ solution (10%) 5 mL L^−1^, MgSO_4_ solution (10%) 2 mL L^−1^, bromothymol blue solution (5%) in KON (2N) 2 mL L^−1^, CaCl_2_ solution (10%) 2 mL L^−1^, micronutrient stock solution 2 mL L^−1^ [per 200 mL micronutrient stock solution: MnSO_4_ 0.235 g, ZnSO_4_·7H_2_O 0.024 g, CuSO_4_·5H_2_O 0.008 g, boric acid 0.280 g, Na_2_MoO_4_·2H_2_O 0.2 g, EDTA solution (1.64%) 0.8 mL, KOH 4.5 g]; and (ii) NLB (mannitol 0.2%, KH_2_PO_4_ 0.1%, CaCl_2_ 0.02%, MgSO_4_ 0.02%, soil extract 10%, NaCl 0.02%, FeSO_4_ 0.0005%). Both media were supplemented with 1% purified agar without N and without any microbial inhibitors. Cultural characteristics were observed for each medium after 2–3 weeks of incubation at 22 °C.

### Siderophore production

Methods described by Alexander and Zubere^47^ were used for determination of siderophore production. Briefly, 250 mL Erlenmeyer flasks containing 50 mL of CAS broth containing soluble starch 10 g L^−1^, vitamin free casein 0.3 g L^−1^, KNO_3_ 2 g L^−1^, MgSO_4_·7H_2_O 0.05 g L^−1^, NaCl 2 g L^−1^, K_2_HPO_4_ 2 g L^−1^, CaCO_3_ 0.02 g L^−1^ and FeSO_4_·7H_2_O 0.01 g L^−1^ were inoculated with a loop full of actinomycete isolates and incubated for 2 days in a rotary shaker at 27 ± 2 °C. After filtering the culture supernatant using a 0.2 μm membrane, 500 μL of the sample was added to an equal amount of CAS broth in an autoclaved Eppendorf tube. The resultant mixture was thoroughly homogenized and observed for change from blue to pink or brown color. Un-inoculated broth was used as a negative control. The production of siderophore was categorized in five scales based on the intensity of the color formation as follows: “−” = no change in color (blue); “+” = light brown; “++” = brown; “+++” = light pink; “++++” = pinkish brown.

### Proteolytic activity

The proteolytic activity of the identified actinomycete isolates was detected using casein starch broth following the procedure of Mohammedin^48^. Briefly, 100 μL suspension of spores from pure cultures were added to 500 mL of caesin starch broth and incubated for 18 h at 37 °C. The cells from 100 mL of culture broth were harvested by centrifugation at 8,000 rpm for 20 min, then washed with SDW. Subsequently, the enzymes were precipitated by slowly adding one volume of ammonium sulphate with constant stirring to give 85% saturation. The precipitated proteins were dissolved in 20 mL of 0.1 M Tris-HCl (pH 7.5) buffer and used as crude enzyme extract for estimation at 280 nm. One unit of proteolytic activity was expressed as the amount of enzymes required to liberate 1 μM of tyrosine per min under the assay conditions.

### Direct anti-mildew activity of proteolytic actinomycetes

Differential influence of actinomycete isolates on *S. graminicola* sporangia formation (%) and zoospore release (%) was analyzed by mixing 500 μL of *S. graminicola* zoosporangium suspension (4 × 10^4^ zoosporangium mL^−1^) obtained from fresh leaves in 500 μL of actinomycete spore suspension, and the anti-mildew activity was assayed as described by Jogaiah *et al.*^49^. SDW treatment of infected leaves (1 cm^2^) was served as control.

### Production of inoculum and seed treatment

#### Inoculum production

Half strength ‘S’ medium was used for growing the actinomycete isolates at 27 °C for 2 to 9 days. The growth was monitored until complete sporulation occurred. Subsequently, the spores and the mycelia were collected into 3 mL SDW with the aid of sterile loop to create a homogeneous suspension. The suspension was then filtered using a double layer muslin cloth, and the spores were counted using a haemocytometer. *Seed-coating with spore solution.* Seeds of HB3 pearl millet cultivar highly susceptible to *S. graminicola* were surface-disinfected with 1.5% sodium hypochlorite for 5 min, then thoroughly washed in SDW. Three mL of the mycelial suspension containing approximately 10^8^–10^10^ spores mL^−1^ of actinomycetes were prepared using haemocytometer. 50 surface-disinfected HB3 seeds were mixed in 1 mL of the prepared mycelial suspension, and were shaken until seeds were fully coated. After 3 h, coated seeds were air-dried and used for further studies under greenhouse conditions. *Seed-soaking with CFE*. 100 mL of casein starch broth were prepared, inoculated with pure culture of actinomycete isolates and incubated for 18 h at 37 °C. The CFEs of the broths were then prepared by centrifugation at 8,000 rpm for 20 min. 50 surface-disinfected HB3 seeds were soaked in 10 mL of CFEs prepared from actinomycete isolates, incubated for 6 h on a rotary shaker and then blot-dried under aseptic conditions. Subsequently, treated seeds were subjected to disease protection studies under greenhouse conditions.

### Efficacy of actinomycete isolates in controlling pearl millet downy mildew disease under greenhouse conditions

To test the efficacy of actinomycete isolates in controlling downy mildew disease, forty susceptible HB3 seeds coated with actinomycete isolates were sown in prepared earthen pots (ten seeds per pot) filled with sand, soil and manure in the proportion of 2:1:1. Four pots were maintained for each actinomycete isolate which were randomly arranged and maintained under greenhouse conditions (day night alternative cycle of 16/8 h, 25/19 °C and 70% relative humidity). HB3 seeds soaked in SDW were served as control. As standard chemical controls, 2.1% of metalaxyl in the form of Apron 35 SD (4 g kg^−1^ seeds) and metalaxyl (350 g a.i. L^−1^; a.i., active ingredient) in the form of Apron XL 350 ES (1.5 mL kg^−1^ seeds) were used for seed-coating and -soaking treatments, respectively. Five-day-old plants were inoculated at the whorl region^50^ (i.e. early two-leaf stage of the cotyledons) with *S. graminicola* zoospore suspension at a concentration of 4 × 10^4^ zoospores mL^−1^ SDW for 3 consecutive days using a capillary dropper. Experimental plants were monitored daily for the expression of typical symptoms. The disease incidence was recorded when the plants were 30-day-old and the percent downy mildew protection was calculated according to Jogaiah *et al.*^51^.

### *In planta* colonization assay

One-month-old plants raised from seeds coated with SJ_UOM-07-09 and 18-09 (10^8^–10^10^ spores mL^−1^) were uprooted completely. Subsequently, the roots and leaves were surface-sterilized with 1.5% sodium hypochlorite for 5 min, washed with 50 mL of SDW for 3 times and dried. Cleaned roots and leaves were then cut into pieces with size of approximately 2 cm and 2 cm^2^, respectively, which were aseptically transferred to plates containing ‘S’ medium. The plates were then incubated at 27 °C until the growth of SJ_UOM-07-09 and SJ_UOM-18-09 occurs.

### Optimization of time required for the proteolytic *Streptomyces griseus* SJ_UOM-07-09 and *Streptosporangium roseum* SJ_UOM-18-09 to reduce downy mildew disease incidence

The 5-day-old plants raised from seeds coated with *Streptomyces griseus* SJ_UOM-07-09 and *Streptosporangium roseum* SJ_UOM-18-09 were inoculated at the early two-leaf stage of the cotyledons with 50 mL of *S. graminicola* zoospore suspension at the concentration of 4 × 10^4^ zoospores mL^−1^ SDW by hand sprayer (i.e. when the plants were 5-, 6-, 7- 8- and 9-day-old after sowing) in different sets of plants. As control, the same conditions were followed for SDW-treated seeds. The pots were maintained under greenhouse conditions in a randomized complete block design as mentioned above. Four pots, each planted with ten coated seeds, were used for each proteolytic isolate. At the end of the 30-day period, disease incidence was counted and the protection rate was calculated as described above.

### Statistical analysis

Data shown are means ± standard errors (SEs) of 4 replications. All the data were subjected to an one-way analysis of variance (ANOVA) using SPSS v. 18.0 (SPSS Japan Inc., Tokyo, Japan). For the assays of *in vitro* proteolytic activity, inhibition of *S. graminicola* growth and the disease protection under greenhouse conditions, the significant differences of treatments were determined according to the Scheffe’s post hoc test (*P* < 0.05).

## Additional Information

**How to cite this article**: Jogaiah, S. *et al.* Isolation and evaluation of proteolytic actinomycete isolates as novel inducers of pearl millet downy mildew disease protection. *Sci. Rep.* **6**, 30789; doi: 10.1038/srep30789 (2016).

## Figures and Tables

**Table 1:** Classification of identified endophytic actinomycete strains isolated from pearl millet roots using *16S* rRNA sequencing and morphological assays.

Group	Subgroup	Strain	Species	Accession number	Size (bp)	Color of aerial mycelia	Pigment	Spore chain
I	I	SJ_UOM-04-09	*Streptosporangium amethystogenes*	KX139494	1100	white to pale pink	+	Spiral
		SJ_UOM-09-09	*Streptosporangium amethystogenes*	KX139495	1423	white to pale pink	+	Spiral
		SJ_UOM-13-09	*Streptosporangium amethystogenes*	KX139496	1233	white to yellowish pink	+	Spiral
		SJ_UOM-39-09	*Streptosporangium amethystogenes*	KX139497	1323	white to pale pink	+	Spiral
	II	SJ_UOM-17-09	*Streptosporangium vulgare*	KX139501	1305	white to orange	+	Spiral
		SJ_UOM-28-09	*Streptosporangium vulgare*	KX139502	1182	orange	+	Spiral
		SJ_UOM-31-09	*Streptosporangium vulgare*	KX139503	1336	yellow to orange	+	Spiral
	III	SJ_UOM-18-09	*Streptosporangium roseum*	KX139499	1377	pale yellow to orange	+	Spiral
		SJ_UOM-34-09	*Streptosporangium roseum*	KX139498	1342	pale yellow to orange	+	Spiral
	IV	SJ_UOM-14-09	*Streptosporangium shengliense*	KX139500	1390	white to yellow	_	Straight
II	I	SJ_UOM-03-09	*Streptomyces griseus*	KX139472	1379	yellowish white	+	Straight
		SJ_UOM-06-09	*Streptomyces griseus*	KX139473	1336	yellowish white	+	Straight
		SJ_UOM-07-09	*Streptomyces griseus*	KX139471	1281	yellowish white to orange	+	Straight
		SJ_UOM-11-09	*Streptomyces griseus*	KX139475	1375	yellowish white	+	Straight
		SJ_UOM-20-09	*Streptomyces griseus*	KX139479	1379	yellowish white	+	Straight
		SJ_UOM-22-09	*Streptomyces griseus*	KX139478	1420	yellowish white	+	Straight
		SJ_UOM-23-09	*Streptomyces griseus*	KX139474	1393	yellowish white to orange	+	Straight
		SJ_UOM-33-09	*Streptomyces griseus*	KX139476	1378	yellowish white	+	Straight
		SJ_UOM-44-09	*Streptomyces griseus*	KX139477	1386	yellowish white	+	Straight
	II	SJ_UOM-01-09	*Streptomyces coelicolor*	KX139483	1434	pale white to yellow	+	Spiral
		SJ_UOM-02-09	*Streptomyces coelicolor*	KX139480	1395	pale white to yellow	+	Spiral
		SJ_UOM-08-09	*Streptomyces coelicolor*	KX139482	1263	pale white to yellow	+	Spiral
		SJ_UOM-12-09	*Streptomyces coelicolor*	KX139486	1357	pale white to yellow	+	Spiral
		SJ_UOM-15-09	*Streptomyces coelicolor*	KX139484	1358	pale white to yellow	+	Spiral
		SJ_UOM-30-09	*Streptomyces coelicolor*	KX139487	1206	pale white to yellow	+	Spiral
		SJ_UOM-32-09	*Streptomyces coelicolor*	KX139485	1373	pale white to yellow	+	Spiral
		SJ_UOM-40-09	*Streptomyces coelicolor*	KX139481	1365	pale white to yellow	+	Spiral
	III	SJ_UOM-05-09	*Streptomyces verticillus*	KX139490	1358	white	_	Straight
		SJ_UOM-10-09	*Streptomyces verticillus*	KX139491	1371	white	_	Straight
		SJ_UOM-21-09	*Streptomyces verticillus*	KX139493	1297	white	_	Straight
		SJ_UOM-24-09	*Streptomyces verticillus*	KX139492	1451	white to pale yellow	_	Straight
		SJ_UOM-25-09	*Streptomyces verticillus*	KX139488	1433	white	_	Straight
		SJ_UOM-45-09	*Streptomyces verticillus*	KX139489	1429	white	_	Straight
	IV	SJ_UOM-27-09	*Streptomyces* sp.	KX139506	1354	white to orange	+	Straight
		SJ_UOM-36-09	*Streptomyces* sp.	KX139505	1273	yellowish	_	Straight
		SJ_UOM-37-09	*Streptomyces* sp.	KX139509	1293	white to yellow	_	Straight
		SJ_UOM-38-09	*Streptomyces* sp.	KX139507	1260	yellowish	+	Spiral
		SJ_UOM-41-09	*Streptomyces* sp.	KX139508	1369	white to yellow	+	Straight
		SJ_UOM-43-09	*Streptomyces* sp.	KX139504	1260	white to yellow	+	Spiral

“+”, produces diffusible pigment; “−”, not produce diffusible pigment.

**Table 2:** Abilities of 36 actinomycete isolates to grow on N-deficient culture media and/or produce siderophores.

Isolates	Growth on N-low medium	Growth on N-free medium	Siderophore production
SJ_UOM-01-09	+	+	−
SJ_UOM-02-09	+	+	+
SJ_UOM-03-09	+	+	−
SJ_UOM-04-09	+	+	+++
SJ_UOM-05-09	+	+	+
SJ_UOM-06-09	−	−	−
SJ_UOM-07-09	+	+	++++
SJ_UOM-08-09	+	+	−
SJ_UOM-09-09	+	+	+++
SJ_UOM-10-09	+	+	−
SJ_UOM-11-09	+	+	−
SJ_UOM-12-09	−	−	++
SJ_UOM-13-09	−	−	+
SJ_UOM-14-09	+	+	−
SJ_UOM-15-09	+	+	+
SJ_UOM-17-09	+	+	−
SJ_UOM-18-09	+	+	++++
SJ_UOM-20-09	+	+	+
SJ_UOM-21-09	+	+	+
SJ_UOM-22-09	−	−	−
SJ_UOM-23-09	+	+	−
SJ_UOM-24-09	+	+	+++
SJ_UOM-25-09	+	+	++
SJ_UOM-27-09	+	+	+
SJ_UOM-28-09	−	−	+
SJ_UOM-30-09	+	+	−
SJ_UOM-31-09	+	+	−
SJ_UOM-32-09	−	−	−
SJ_UOM-33-09	+	+	++
SJ_UOM-34-09	+	+	+
SJ_UOM-36-09	+	+	+++
SJ_UOM-37-09	+	+	−
SJ_UOM-38-09	−	−	+
SJ_UOM-39-09	+	+	+
SJ_UOM-40-09	+	+	+
SJ_UOM-41-09	+	+	−
SJ_UOM-43-09	+	+	++
SJ_UOM-44-09	+	+	−
SJ_UOM-45-09	+	+	+++

“+”, actinomycete isolates either show growth on N-deficient media or produce siderophores; “−” absence.
